# Rethinking Middle Management in Residential Aged Care: Evaluation of a Dyad House Leadership Model Intervention

**DOI:** 10.1155/jonm/3098235

**Published:** 2026-09-18

**Authors:** Katrina Radford, Ellie Meissner, Michelle Church, Jenna Maddern, Demi Perkins

**Affiliations:** ^1^ Department of Management, Griffith University, Brisbane, Australia, griffith.edu.au; ^2^ School of Psychology, University of Southern Queensland, Ipswich, Australia, usq.edu.au; ^3^ St Basil’s Homes for the Aged in SA (Vasileias) Inc, Adelaide, Australia

**Keywords:** aged care workforce, career pathways, dyad leadership, middle management, mixed methods, pre–post intervention

## Abstract

**Background:**

New leadership models in Australian aged care facilities are required to respond to increasing demands, decreasing supply of talent and more complex care clients.

**Aim:**

In this study, we cocreated and evaluated a dyad house leadership model across three residential aged care sites in South Australia using a pre–post intervention methodology.

**Methods:**

Data were collected over three time periods. We first interviewed employees (*n* = 28), families (*n* = 8) and residents (*n* = 8), then conducted 90 h of observations across 9 leaders. Three codesign sessions were then held to cocreate the solution. The intervention (a dyad house leadership model) was trialled for 6 months. We then evaluated by conducting interviews with employees (*n* = 21 mid and *n* = 28 post), families (*n* = 6 mid, and *n* = 8 post) and residents (*n* = 8 mid and *n* = 13 post) and observations with leaders (*n* = 32 h mid, *n* = 96 h post).

**Results:**

Participants reported improved role clarity, career progression, wellbeing and perceptions of quality of care alongside greater satisfaction with communication and care; however, some clinicians perceived some risks to their career progression. At the 6‐month point, no significant changes were found in the counts of hospitalisation, client incidents and average call‐bell response time.

**Conclusion:**

Dyad house leadership represents promising middle‐management model for residential aged care, with potential to improve role clarity, redistribute operational workload and expand leadership capacity. Successful implementation requires clearly defined clinical and nonclinical accountabilities, and careful attention to clinical governance and succession planning.

**Implications for Nursing Management:**

Effective dyad leadership requires both deliberate career infrastructure and structures that enable clinical and nonclinical leaders to work together effectively. Clear progression pathways, reporting relationships, role transitions and shared ways of working can help broaden the leadership pipeline while preserving clinical career trajectories and reducing traditional silos.

## 1. Introduction

Leading healthcare organisations post‐COVID‐19 requires innovation, teamwork and dynamic teams [1]. Yet the appetite for such innovation is often met with blockages and rigid thinking around clinical and nonclinical leadership models in aged care homes. For example, the Royal Commission into Aged Care acknowledged the important role that leadership plays in aged care outcomes; however, recommendations remain narrowly focused on clinical leadership models [2]. This reflects a broader pattern in aged care, where leadership has traditionally been conceptualised as the responsibility of clinicians, despite growing complexity of organisational and relational challenges that extend beyond clinical boundaries.

The importance of nurse leadership has been highlighted as critical in reducing the moral distress experienced within nurses [3] and in improving staff wellbeing and reducing staff turnover [4]. However, evidence‐based alternatives to clinical‐only leadership remain limited while care complexity, expectations of care from family members and clinical vacancies across aged care increase [2, 5]. A broader conceptualisation of leadership that draws on both clinical and nonclinical expertise may, therefore, be necessary to address persistent workforce and organisational pressures [6]. One possible solution to this challenge is the introduction of a new dyad house leadership model.

A traditional dyad leadership model pairs a clinician leader and a nonclinician leader in a team situation [7]. Definitively, dyads are mini teams of two people who work together as coleaders of a specific system, division, clinical service line or project [7]. Our study extends this further by using a dyad house leadership model, which refers to a middle‐management structure in which a specific area of a residential aged care facility is coled by a clinical and nonclinical leader. The clinical leader is responsible for all clinical decisions within the house, whereas the nonclinical leader maintains responsibility for all nonclinical decisions (such as family communication, lifestyle/leisure activities, personal care work, hospitality and laundry and maintenance issues). In the dyad house leadership model, we also ensured the same regular staff worked within each house and reported to the relevant leader, with personal care workers, lifestyle/leisure, maintenance and hospitality staff reporting to the nonclinical leader, and registered nurses (RN), enrolled endorsed nurses (EEN) and enrolled nurses (EN) reported to the clinical leader. The clinical leaders all reported to the care manager and the nonclinical leaders reported to the general manager. The household structure locates leadership closely to residents, families and frontline staff and embeds clinical and operational accountability within the same local unit. Importantly, the model creates a career infrastructure around the dyad by establishing distinct clinical and nonclinical leadership pathways, reporting relationships and progression opportunities.

This extends dyad leadership beyond pairing of two individuals to the existing organisational structure that supports those roles [7]. This is theoretically distinct from distributed leadership [8, 9], which is focused on mobilising leadership throughout multiple people at the same level (sometimes termed collective leadership) or where different units collaborate on plans, decisions and actions, without distinct portfolios (sometimes termed coleadership). It is also distinct from shared leadership, which emphasises collective influence, whereby leadership emerges from reciprocal interactions among team members rather than leadership being tied to formal roles [10].

Another important distinction in the dyad house leadership model is the emphasis on the dyad house leadership roles being in middle management positions reporting to the residential aged care manager within residential aged care facilities. Middle management in health care are key players for organisational change, translating clinical governance expectations into operational routines, human resource management, resident experiences, communication with stakeholders and driving quality care outcomes [11–13]. Middle managers are also seen as the operational boundary‐spanners who manage the interface between clinical requirements and nonclinical service delivery. We, therefore, argue that middle management is an important intervention point in residential aged care because these roles operate at the interface of clinical governance, frontline service delivery and organisational management. A dyad structure at this level may allow operational issues to be addressed closer to the point of care while enabling residential managers to retain greater strategic oversight.

The implementation of a dyad leadership model in healthcare fields has emerged progressively in healthcare organisations over the past 15 years [14]. A narrative review of studies exploring dyad leadership models more broadly found only six quality papers that explored the impact of this model in hospitals and called for more rigorous studies examining the impact of this model on quality and patient outcomes [15]. Recently, a mixed methods study by Saxena [14] found that to be successful, dyad leadership models must seek to address interpersonal concerns around mindset, competencies, relationships and communication along with allocating time, support and investing in collaboration through joint accountability. Similarly, a qualitative study comparing American and Australian hospital leadership strategies found that dyad leadership models overcame communication issues and provided better healthcare outcomes for patients [16]. However, most of the dyad leadership teams evaluated included physicians and nurses, or executive and clinical leadership models in an acute setting.

Existing dyad leadership research, therefore, leaves three questions unresolved. First, it is unclear whether dyad leadership structures translate effectively from acute care to residential settings characterised by long‐term relationships with residents and families. Second, little is known about dyads positioned specifically at the middle‐management level in residential aged care. Third, previous studies have paid limited attention to how dyad structures interact with career pathways, professional identity and professional boundaries.

Given the aged care sector requires both clinical governance and operational management, we argue that the extension of the traditional dyad leadership model to a dyad house leadership model might be an effective opportunity to formalise collaboration without diluting accountability. This allows the positions to complement but not replace distributed or shared leadership approaches as relying on clinical governance or operational management alone might undermine care quality or organisational sustainability.

However, no study globally has examined the impact of a clinical and a nonclinical leadership dyad in middle management positions within residential aged care settings. This study becomes the first to report on these outcomes by exploring the overarching research question:

*How does a dyad house leadership model impact staff, family and residents who work, visit and live in residential aged care?*

## 2. Methodology and Methods

### 2.1. Design

This study employed a longitudinal, qualitatively driven mixed‐method design to evaluate the implementation of a dyad house leadership model across three residential aged care sites. Both qualitative and quantitative data were collected over three time points, with the same people where possible, within the same organisation. Data were collected before, during and following implementation from staff, residents, family members and organisational leaders. Qualitative interviews and focus groups constituted the primary source of evidence, complemented by structured observations of leadership time use and organisational indicators relating to hospitalisations, client incidents and call‐bell response times. The findings from these data sources were analysed and interpreted together to examine whether participants’ experiences were consistent with observed changes in leadership work and organisational indicators at each time point. The pre–data collection (interviews and observations) was interpreted together and presented as a starting point in the codesign sessions, the midpoint data were then collected at one site to minimise impact and analysed before the final data collection was conducted and the findings interpreted as a whole. The quantitative data were then compared to the qualitative data (interviews and observations) at each stage and again at the end to evaluate the wider impacts of the model implementation.

### 2.2. The Intervention

The intervention involved the codesign and implementation of a dyad house leadership model supported by a revised role matrix. Each residential house was jointly led by a clinical care coordinator and a living well house manager, who held distinct but complementary clinical and nonclinical portfolios. Clinical governance and clinical decision‐making remained with the clinical leadership, while the nonclinical leadership pathway incorporated looking after family communication and day‐to‐day household operations, supported by lifestyle, care and hospitality staff. Reporting relationships and role boundaries were formalised through the role matrix. Codesign and training workshops preceded implementation, after which the model was trialled for 6 months.

### 2.3. Participants and Recruitment

Participants were recruited from three residential aged care facilities operated by the participating organisation. Residents were eligible if they (1) were a permanent resident of one of the facilities, (2) could provide informed consent and (3) had sufficient English language proficiency to undertake an interview or access to an interpreter. Family members were eligible if they (1) had a family member living at one of the participating facilities, (2) could provide informed consent and (3) participate in an interview in English. Staff were eligible for interview if they (1) were employed by the organisation during the study period, (2) could provide informed consent and (3) participate in an interview in English. Staff participating in observations were additionally required to hold a leadership role at one of the three sites and agreed to be shadowed by a researcher. Recruitment occurred through facility communications, staff invitations and researcher presence on‐site. Purposive and snowballing sampling were used to include relevant participant groups across participating facilities.

Ethical approval was obtained in two stages from REDACTED. The first ethical approval was obtained to conduct the pre–data collection and codesign phase. The second ethical approval was obtained once more information was known about the intervention and subsequent data collection methods. This strategy was chosen in order to not hold up the project. Consent to participate in the research was provided in writing and/or verbally (if the interview was conducted online, or if the interview was the second/third conducted by the researcher) by all participants. Consent to record the interview was provided as a secondary layer with two staff members refusing to allow the interview to be recoded, but who were happy to participate in the interview. In these instances, copious amounts of notes were taken during the interview to ensure it was accurately captured. To manage the risk of the organisation forcing participants to talk to us, we provided each participant an explicit invitation to withdraw from the study if they did not willingly volunteer without the risk of disclosure to their employer. This strategy resulted in two participants removing themselves from the research study. Data are stored securely in a locked online file accessible only by the researchers involved in the study. Finally, the researcher’s email was sent to all families, residents and staff members with an open invitation to report concerns about the model along the way, to manage the risk of poor care outcomes because of the trial. These risks were then raised in our weekly project meeting.

### 2.4. Data Collection

#### 2.4.1. Pretrial Data Collection

Between May and October 2023, interviews were conducted with 28 staff members (95% female, 5% male), eight family members (20% male, 80% female) and eight residents (50% male, 50% female) who lived in one of the three facilities operated by the organisation. Half of the residents elected to have an interpreter present; no family or staff needed an interpreter present. Nine leadership staff (77% female, 23% male) were shadowed for approximately 10 h each to characterise leadership time use during a typical workday in aged care. Following initial analysis, three codesign sessions were held over a 3‐week period, to reflect on the findings identified and to cocreate the solution. These sessions lasted on average 3 h each and were attended by eight leadership staff representing each facility.

All observations focused on the time taken and tasks undertaken at each time point. Interview questions focused on their perceptions of client care, satisfaction with their jobs, pain points with their jobs and areas for improvements. The results of all predata then informed the codesign process.

#### 2.4.2. Codesign and Implementation Preparation

The codesign sessions were then implemented to identify opportunities to improve work‐load distribution, efficiencies and redesign the role scopes for each role within the organisation on site. Consideration was given to ensuring that clinical governance was maintained, leadership and business activities were accounted for and new management lines for care and lifestyle staff as well as clinical staff were implemented. Resident and family feedback from the interviews were incorporated into the codesign problematisation but only staff were included in the codesign workshops as the intervention was targeted at staff roles redesign. At the completion of the codesign sessions, staff were then provided access to the new role matrix, which held their new accountabilities and role boundaries, for 3 weeks. Following codesign, staff were given 3 weeks to review the revised role matrix and proposed accountabilities before implementation. This period was determined by the participating organisation as sufficient for consultation and familiarisation. Following this process, training sessions were conducted at each site to explain the model prior to implementation. The model was then implemented for 6 months. Weekly clinical governance meetings were maintained throughout implementation to monitor resident safety, clinical accountability and care quality.

#### 2.4.3. Mid Trial Data Collection

The mid‐point data collection was conducted in March 2024 at only one site and comprised of 21 staff members (18 females, 3 males), 6 family members (1 male, 5 female), 8 residents (6 female, 2 male) and 32 h of observations of 4 key leadership positions at one site. No staff, resident or family member needed an interpreter present. Due to resource constraints, mid‐trail observational data were collected at only one of the 3 sites. The observations included one RN, one clinical care coordinator, one care manager and one living well house manager. A follow‐up focus group was then conducted with 8 senior leaders to reflect on the findings and provide further insight on their experiences at this point of the trial. This focus group lasted 2 h and was conducted in person at one site, with all except one staff member attending in person.

#### 2.4.4. Posttrial Data Collection

The final data collection was conducted between May and July 2024. This consisted of 28 staff members (25 female, 3 male), 13 residents (3 male, 10 female) and 8 family members. Posttrial observation totalled 96 h across 12 leadership staff; most were observed for approximately 8 h, with shorter observations for two participants due to operational availability. No staff or family member needed an interpreter present. However, 2 residents had 1 staff member available to interpret if needed. Interviews with residents lasted between 8 min and 1 h. Interviews with families lasted between 20 min and 1.5 h. Interviews with staff lasted between 20 min and 1 h. Staff interviews were collected with the same personnel where possible (GMs); however, some turnover had occurred in the nursing positions (CL and CM) and one of the NCL roles during the project. In addition, whilst the project set out to interview the same family and residents across time, natural turnover within the facility made this impossible. Therefore, no within‐person effects can be interpreted from the resident and family interviews. Please note: The observations were collected with the same personnel where possible (GMs); however, some turnover had occurred in the nursing positions (CL and CM) and one of the NCL roles during the project and as such care should be taken when interpreting these results.

#### 2.4.5. Interview Guide

Semistructured interview guides were tailored to the participant group. Residents and family interviews explored experiences of staffing, communication, lifestyle activities and perceived quality of care, including areas for improvement. Staff interviews explored career experiences, daily work activities, workload challenges, perception of resident care and opportunities for service improvement. Questions did not explicitly prompt participant to comment on workforce capacity or the dyad model, allowing relevant issues to emerge from participants’ accounts. The same interview guides were used across pre‐, mid‐ and postintervention data collection points to support consistency in the topics explored over time. Interviews were conducted by experienced qualitative researchers (Authors 1 and 2).

#### 2.4.6. Observations

Structured observations recorded time allocated to direct resident contact and resident‐related duties, interaction with family members, documentation and email, staff support, meetings, training and breaks. Duration was recorded in minutes using a standardised observation template. Researchers did not enter confidential meetings; where this occurred, meeting duration was recorded without documenting content. The observations were performed by a clinical researcher. Appendix 1 presents the template used to capture these data. Please note: The observations were collected with the same personnel where possible (GMs); however, some turnover had occurred in the nursing positions (CL and CM) and one of the NCL roles during the project and as such care should be taken when interpreting these results.

#### 2.4.7. Quantitative Data

This study compared the pre–post scores across the average response to call bells, hospitalisation rates and number of client incidents across all three sites with the same period the year before. This means that we compared the data obtained from December 2022 to May 2023 with the trial period data between December 2023 and May 2024. This was done to try to control the seasonal differences when comparing data immediately prior and after the interventions. We did not compare at a site level because the small number of identifiable facilities could compromise organisational confidentiality.

### 2.5. Data Analysis

#### 2.5.1. Qualitative Analysis

Transcripts and field notes were analysed using reflexive thematic analysis following Braun and Clarke’s approach [17–20]. Analysis was primarily inductive and involved iterative familiarisation with the data, coding, development and refinement of candidate themes and interpretation of patterns of shared meaning across the dataset. The two lead researchers initially engaged with different components of the dataset and met regularly to critically discuss their interpretations, consider alternative readings and refine the developing thematic analysis. One lead researcher initially focussed on the resident and family dataset, while the second focused on staff data. The researchers brought this analysis together through iterative reflexive discussion to examine patterns of convergence, divergence and complexity across participant groups and time points. These discussions were used as a reflexive process to deepen interpretation and support reflexive rigour rather than to establish coding consensus or interrater agreement. Regular reflexive discussions and peer debriefing with clinical and nonclinical project partners were used to challenge assumptions, consider alternative interpretations and strengthen the depth of the analysis. Consistent with reflexive thematic analysis, the researchers recognised their active role in knowledge production and used ongoing reflexive discussion to consider how their disciplinary backgrounds, assumptions and involvement in the project shaped interpretation.

#### 2.5.2. Observational Analysis

Observational data were summarised descriptively, with time allocated (e.g., direct resident contact, documentation and meetings) collated in Microsoft Excel to allow a comparison across time points. Quantitative indicators (hospitalisation, call‐bell response times and incidents) were analysed using paired samples *t*‐tests to compare outcomes between pretrial and trial periods. Given the small sample size and organisational‐level analysis, the findings are presented descriptively alongside qualitative insights to provide triangulation rather than definitive causal inference.

#### 2.5.3. Organisational Indicators

Hospitalisation, client incidents and average call‐bell response time were analysed using paired‐samples *t* tests to compare the preintervention across all facilities with the same time point post the intervention period. These data points were gathered by the site’s quality officer and reflected real‐time data captured by the organisation and reported internally to the board. Effect sizes were calculated to assist interpretation of the magnitude of observed differences. Given the small number of paired observations, statistical findings were interpreted cautiously and considered alongside the qualitative and observational data.

#### 2.5.4. Integration of Findings

Qualitative, observational and organisational findings were integrated during interpretation to examine areas of convergence, divergence and complementary across data sources. The observational and organisational data were used to contextualise and extend participants’ accounts, rather than as standalone evidence of causal effects.

## 3. Results

### 3.1. Role Clarity and Workload

At the start of the trial, participants perceived high levels of job satisfaction (“I love the work I do”) but also heavy workloads, administrative burden and reactive task management (“*Workload is the number one dislike for me. I enjoy the type of work but not the volume. Sometimes it requires long hours and weekends to meet the deadlines*”), reactive workloads (“*It’s really just responding to whatever is required at the point in time*”) and a desire to be more person centred in their approach to work (“*Id like more time on the floor. The downside to this is less time with your team that you manage so it become(s) difficult to manage*”).

At the midpoint of the trial, the shifts were starting to show at the site level as staff began working in the house area, yet employees still felt reactive in their work tasks and were unconvinced about the impact of the model. For example, one staff reported: *“Like every day is the same thing. I haven’t seen any changes or different.”* Where other staff members did feel the change but were still relatively resistant to the nonclinical leader role. Upon unpacking this resistance, it became clear that some boundary issues had emerged between the clinical and nonclinical staff. For example, one clinical leader reported*, “So sometimes you have to override (non-clinical leaders’) decision…, because end of the day for me, resident integrity is key. And you know, it is my duty of care to make sure their safety.”*

Debriefing with leaders across all sites indicated a sense of cautious optimism. A senior clinical leader noted: “*For us the positives have been the improved sense of teamwork, but we risk being fractured from other teams. We’ve been so busy with departures and admissions and workload pressures… you get into your own thing, but without that connection the risk is of being fractured*.” Similarly, a nonclinical leader observed: “*My experience has been positive… we’re able to do much more than before, and our floor time increased significantly. The extra floor time is great, but it is at the detriment of administration, and I’m falling behind there*.” From a corporate perspective, the workforce lead reflected: “*We have felt positive effects over the role clarity and seen really good outcomes*.” Collectively, these reflections led to the decision to extend the trial for an additional 3 months.

By the end of the trial, staff perceived greater clarity and better distribution of tasks: *“Overall, this model has allowed us to distribute work evenly and spend more time with residents.”* This was also evidenced in the observational data (Table 1).

**TABLE 1:** Comparison of daily time allocation across leadership groups during the trial (minutes per day).

Activity	GM pre	GM post	NCL pre	NCL mid	NCL post	CM pre	CM mid	CM post	CL pre	CL mid	CL post
Direct contact with residents and duties performed	13.0	10.5	99.0	115.0	122.0	49.0	3.0	29.5	122.5	12.0	77.0
Time with family members	9.5	9.5	8.33	142.0	26.5	17.5	6.0	7.5	12.0	7.0	12.5
Documenting and responding to emails	109.0	117.0	201.67	77.0	81.6	205.0	15.0	213.5	226.5	147.0	127.0
Breaks	14.0	37.0	4.33	31.0	20.0	10.0	11.0	18.5	33.5	55.0	54.0
Time with staff	68.5	161.0	51.33	143.0	94.0	213.0	95.0	121.0	37.5	154.0	159.5
Meetings	251.0	68.0	71.33	13.0	26.75	40.5	71.0	33.0	25.0	0.0	0.0
Training	N/A	N/A	N/A	N/A	N/A	0.0	105.0	0.0	N/A	N/A	N/A

*Note:* Values represent average minutes spent per day on each activity during observation periods. Values are descriptive averages based on observed leadership time use. For general managers, many activities previously classified as “meetings” were conducted on the floor and focused on staff engagement. Consequently, the observed reduction in meeting time (251–68 min) coincided with a substantial increase in time spent with staff (68.5–161 min). Mid trial data points were collected from one site only so care should be taken when interpreting these results.

Abbreviations: CL = clinical lead, CM = care manager, GM = general managers, NCL = nonclinical leaders.

Across leadership groups, the observational data indicated shifts in how work time was allocated (see Table 1), although the direction and magnitude of change differed by role. The largest observed changes included increased resident contact among nonclinical leaders (+23 min), reduced documentation and email time among nonclinical leaders (−120 min), increased staff‐facing time (+92.5 min) and reduced meeting time among general managers (−183 min). Clinical leaders also spent more time supporting staff (+122 min) and less time on documentation and email (−99.5 min). In contrast, care managers spent less time with staff (−92 min) and slightly more time on documentation and email (+8.5 min). Whilst these results should be interpreted with care as they are not all within person results, they do provide an indication of the changes experienced across roles.

Moreover, the interviews also captured the perceived shift in work‐life balance from senior staff, who reported not taking work home as often as previously, where a general manager reflected, “*Overall, this model has allowed us to distribute work evenly and allowed staff to spend more time with residents and to focus on providing quality person-centred care. It has allowed me to empower my leaders to step up and I’m now able to be more strategic and less operational in my role, which contributes to the broader organisation more. We are taking less work home, and staff, families and residents all seem more satisfied*.”

### 3.2. Staff Wellbeing and Professional Identity

At baseline, staff expressed a strong commitment to their roles but also reported emotional strain and exhaustion. Many described their work as reactive and overwhelming: (“*It’s really just responding to whatever is required at the point in time*”). Several also spoke of the toll they felt around the constant administrative demands, limited time for breaks and the need to take work home: “*Workload is the number one dislike for me … sometimes it requires long hours and weekends to meet the deadlines*.” These accounts reflected a workforce motivated by residents’ care but struggling with unsustainable expectations.

By mid‐trial, staff reported that some of these pressures had begun to shift. Staff perceived greater presence of leaders on the floor and stronger collaboration between the clinical and nonclinical managers. However, this redistribution of work created tension. A nonclinical leader commented: “*Our floor time increased significantly, but I am falling behind on administration*”. Others described the model as positive but still in transition, with workloads unevenly balanced. Focus group reflections echoed this mixed picture: Some leaders valued the improved sense of teamwork, while others worried about fragmentation from other teams under the pressure of admissions and departures. At the corporate level, the workforce lead also reflected that “*we have felt positive effects over the role clarity and seen really good outcomes*.”

By the end of the trial, many staff described improvements in wellbeing and balance. Leaders reflected that they were taking less work home, had more manageable workloads and were better supported in their roles. A RN reflected: “*Now, I have support from a clinical nurse, care manager and residential manager. Things are very good*.” A shift in attitude was also reported by a clinical leader we interviewed, who at the beginning of the trial, were resistant to the model, whereas by the end of the 6‐month trial they reflected, “*I think it’s such a great support to the clinical team to have that living well house manager, you know, following up those things that can take the clinical nurse or even myself away from, you know, the clinical things that are needed*”. Another senior leader noted the benefit of being able to focus more strategically: “*I am now able to be less operational in my role, which contributes more broadly to the organisation.*” These accounts suggest that the dyad house leadership model was perceived to alleviate some stressors that had previously undermined staff wellbeing.

Yet alongside these benefits, the introduction of nonclinical leadership roles created concerns about professional identity and succession. One clinician perceived their authority had been eroded: “*It’s almost like the non-clinical leadership position is a step above … I feel like I have half a role now.”* Others questioned whether advancement opportunities for clinicians were being curtailed: “*I must move from my clinical role to the non-clinical role to make it to General Manager.”* These accounts highlight the paradox of the dyad house leadership model. While it offered practical relief and improved wellbeing, it also unsettled established hierarchies and professional identities about the future of clinical leadership.

### 3.3. Residents and Family Experience

At baseline, residents described mixed experiences of care quality, with some dissatisfaction around staff turnover and responsiveness. “*The carers are the ones who make it or break it. They will influence whether you like it here or you don’t. We have three or four carers who have been here a long time they’re great. But the new ones just don’t pick up on the little things and it’s the little things that are the difference between “this is a nursing home or this is your home*.*” Furthermore, another resident responded, “I have also given written feedback to the kitchen about the food. The chef avoided me for a few weeks after that.”* Families reported similarly, noting variable communication with staff and limited proactive updates about care prior to the intervention*. “Communication with dad is limited as I don’t know who to phone or where to go and “if” nurses pick up phone when call*.*”*

By mid‐trial, residents described greater consistency in staff presence within the house model, although some remained unsure whether these changes would be sustainable. Families described greater access to managers and increased visibility of leadership on the floor. However, experiences remained varied, with not all residents engaging directly with activities or staff, reflecting different levels of personal preference.

By the end of the trial, residents and family members described several positive changes in their experience of care. One resident noted: “*The people here now are good before not good … Staff are nice and there is an improved response to call bells*.” Another described feeling freer and more supported: “*I love the freedom I get here … Staff are happy to give me what I need, and I recognise familiar faces now.”* Families also reported marked improvements in communication: One family member reflected, “*In 5 years here, I had never received an email. Now I can email and get a response straight back.”* These accounts suggest improved perceptions of communication, continuity and confidence in care among family members.

### 3.4. Organisational Indicators

Quality of care in aged care is a multifaceted issue that involves various elements such as resident satisfaction, safety and health outcomes. Organisational indicators were examined to explore whether changes in resident‐related outcomes were observed during the intervention period. While these data points should be interpreted with care, the results provide a level of description of the wider impact that the model may have to the organisation. Paired‐samples *t*‐tests were conducted to examine changes in response times for call bells, hospitalisations and client incidents following the intervention. No statistically significant differences were observed for call bells, *t* (2) = −0.31, *p* = 0.786, hospitalisations, *t* (2) = 0.31, *p* = 0.789, or client incidents, *t* (2) = 1.27, *p* = 0.331. Although client incidents demonstrated a moderate effect size (*d* = 0.73), no statistically significant differences were identified. Descriptively, all three organisational indicators decreased during the intervention period (Table 2).

**TABLE 2:** Comparison of key outcome measures before and after the intervention.

Outcome measure	Preintervention (Dec 2022 to May 2023) M	Postintervention (Dec 2023 to May 2024) M	Change
Hospitalisations	36.00	33.33	−2.67
Client incidents	477.33	430.33	−47.00
Average response time to call bells (seconds)	260.25	246.25	−14.00

*Note:* Negative values indicate a reduction from the preintervention period to the postintervention period.

There were two possible reasons for the lack of significant differences identified in this data set. First, it could reflect the acuity of clients changing during this time or it may be too early to measure the impact of these measurements on the intervention. Follow‐up review of the data is recommended in 12 months to examine this further.

### 3.5. Unintended Consequences and Organisational Impact

While the dyad house leadership model was generally well received, participants also identified unintended challenges associated with its implementation. At baseline, leaders reported that the introduction of the dyad house leadership model was a potential way to address persistent workforce shortages and role ambiguity. By mid‐trial, reflections revealed boundary issues between clinical and nonclinical leaders, with some clinicians expressing concerns about decision‐making authority and future clinical career progression.

At the organisational level, senior leaders perceived the model as creating opportunities to broaden the potential recruitment pool, leadership pipeline and enhancing role clarity. One senior manager reflected: “*You’re broadening the workforce by tapping into people who may not have considered aged care … and reducing turnover because you’ve actually got leaders who care*.” These accounts illustrate an important tension within the model: while leaders perceived potential benefits for workforce flexibility and leadership recruitment, some clinical staff experienced uncertainty about professional identity, decision‐making authority and future career progression. This suggests that implementation of the model requires clear governance and career pathways for both clinical and nonclinical leaders.

## 4. Discussion

This study examined the implementation of a dyad house leadership model across three Australian residential aged care homes. Overall, the findings suggest that the model was associated with clearer role differentiation, redistribution of some leadership work, greater leadership visibility and more positive perceptions of communication among staff, residents and families. At the same time, implementation generated tensions relating to professional identity, clinical authority and career progression, highlighting the importance of governance and career infrastructure in supporting dyad leadership.

Through the integration of pre‐, mid‐ and postintervention interviews, focus groups, observations and organisational indicators, to our knowledge, this study represents the first known evaluation of a dyad house leadership model within an Australian residential aged care context. Overall, the findings support previous research conducted in acute healthcare settings by Saxena et al. [14, 21], Leach et al. [16] and Clouser et al. [15]. Like these studies, participants reported improvements in communication, collaboration, role understanding and leadership responsiveness. Saxena et al. [14, 21] identified that dyad leadership structures enhance organisational effectiveness through complementary expertise, shared accountability and improved decision‐making. Consistent with these findings, staff in the present study described clearer delineation of responsibilities between clinical and nonclinical leaders, alongside perceptions of more efficient problem‐solving and reduced duplication of effort. Likewise, Leach et al. [16] reported that collaborative leadership structures strengthened staff engagement and organisational performance. Similar outcomes were observed in this study, with staff reporting increased leadership visibility, improved support and greater confidence in local decision‐making.

Importantly, while this study highlighted significant gains embedding a dyad house leadership model, the findings also highlight potential challenges, or a ‘dark side’, associated with these models. This study found the model challenged existing professional identity and career progression opportunities for clinicians and generated questions of clinical authority. These challenges are similar to those recently reported by Saxena and Yao [22] who argued the pitfalls are mostly due to mental frames and perception of power within the organisation, suggesting that clear communication and organisational structural policies are critical to the smooth implementation of the model. In addition, this study supports and extends both Saxena and Yao [22] and Linhardt et al. [23] calls for a focus on team development both for interprofessional and intraprofessional teams for ongoing harmony.

This study also extends the existing literature by identifying several mechanisms through which our dyad house leadership operates specifically within residential aged care. Unlike acute care settings, residential aged care is characterised by long‐term relationships between staff, residents and families, with quality of life and continuity of care being central outcomes. Consequently, the effectiveness of the dyad model appeared to operate not only through operational efficiency but also through enhanced relationship‐building and household‐level collaboration. The increased visibility of leaders within households created opportunities for informal communication, real‐time problem solving and stronger partnerships with residents and families. These findings suggest that dyad leadership in aged care may be particularly relevant in aged care because it has potential to simultaneously support both, the operational and relational dimensions of care delivery.

Furthermore, this study extends current knowledge by highlighting the impact of dyad house leadership on staff wellbeing, workload management, job satisfaction, work‐life balance and perceptions of care quality. The model facilitated a clearer distribution of responsibilities and increased leadership presence within households. Observational data were broadly consistent with these accounts, showing shifts in time allocation across leadership roles, including less time in meetings for some roles and greater time supporting staff or engaging with residents. These findings align with Saxena et al. [21], who argued that complementary leadership partnerships improve organisational effectiveness by allowing leaders to focus on their areas of expertise while sharing accountability for outcomes. Importantly, however, the present study found that these benefits were not immediately recognised by all staff. Mid‐trial reflections indicated that some participants continued to perceive their work environment as reactive rather than proactive. This suggests that the effectiveness of dyad leadership is dependent not only on structural redesign but also on sufficient implementation time, organisational reinforcement and staff adaptation.

A particularly important contribution of this study is its identification of role clarity as a critical mechanism underpinning successful dyad leadership. While previous dyad leadership studies have acknowledged the importance of clearly defined responsibilities, role clarity emerged in this study as a prominent mechanism associated with staff confidence, accountability and interdisciplinary collaboration. Research on the broader social sector revealed that role clarity by coworkers and leaders play a critical role in the overall wellbeing of workers [23–25]. In this study, similar results were identified as participants consistently reported that clearer leadership responsibilities reduced duplication, improved communication and enhanced decision‐making processes. The findings, therefore, suggest that role clarity is not merely an implementation consideration but a fundamental mechanism through which dyad leadership generates positive outcomes in residential aged care settings.

The dyad house leadership model was also associated with reductions in stress, improved work‐life balance and increased strategic capacity among senior leaders, supporting broader evidence linking supportive leadership structures with staff wellbeing and workforce retention [4]. However, the introduction of nonclinical leadership positions also generated concerns among some clinicians regarding professional identity, clinical authority and future career progression. These findings align with Clouser et al. [15], who noted that shared leadership arrangements can create uncertainty regarding authority and governance if roles are not clearly negotiated and communicated. However, the present study extends Clouser et al.’s findings by demonstrating how these concerns can influence perceptions of workforce pipelines within residential aged care. Some participants questioned whether the introduction of nonclinical leadership roles might reduce opportunities for nurses seeking advancement into management positions. These findings suggest that dyad leadership requires not only clear role design but also deliberate career infrastructure. Organisations implementing similar models should articulate clinical and nonclinical progression pathways, reporting relationships and succession arrangements so that workforce diversification does not inadvertently weaken perceived clinical career opportunities.

Residents and families reported clearer communication, more consistent staff presence and greater trust in care, particularly by the conclusion of the trial. These findings align with previous research highlighting the relational benefits of leadership models that increase visibility, accountability and responsiveness [26]. However, the present study provides new insight into how these benefits manifest within household models of residential aged care. Unlike acute care settings, where interactions are often episodic, residents and families in aged care develop long‐term relationships with staff and leaders. The increased accessibility of both clinical and nonclinical leaders enabled concerns to be addressed more promptly and fostered stronger levels of trust and confidence. These findings suggest that evaluations of leadership interventions in aged care should extend beyond operational indicators to include resident and family experiences as key measures of success.

At an organisational level, the dyad house leadership model broadened the recruitment pool by creating nonclinical leadership pathways, an important consideration given ongoing national aged care workforce shortage [27]. Leaders also reported improved role clarity, increased job satisfaction and reduced turnover intentions. The model, therefore, offers a potential strategy for diversifying leadership recruitment while supporting workforce sustainability. However, concerns regarding role boundaries and clinical authority indicate that governance structures remain critical to successful implementation. Baker’s [28] cooperative problem‐solving framework suggests that shared accountability, negotiation of responsibilities and collaborative decision‐making are essential for partnership‐based leadership models. The present findings reinforce this proposition and demonstrate the need for governance frameworks that clearly define decision‐making authority while protecting clinical accountability.

Finally, this study provides important insight into the role of time in workforce transformation and leadership intervention research. A notable finding was that a 3‐month intervention period was insufficient for participants to fully experience or recognise the benefits of the new leadership model. Even at 6 months, some outcomes appeared to be continuing to emerge. One plausible explanation is that acceptance of workforce redesign occurs before measurable benefits are realised. This interpretation is consistent with Hagemann and Cechlovsky’s [29] change curve model, which identifies four phases of organisational change: unawareness and denial, discomfort and resistance, exploration and discovery and integration and commitment. Evidence from this study suggests that many participants remained within the first two phases during the 3‐month midpoint assessment, whereas by 6 months most had progressed to the exploration, discovery, integration and commitment stages. This finding has important implications for future workforce intervention studies, suggesting that leadership interventions should be evaluated over a longer time to see embedded changes and impacts to the model at a cultural level. Such an approach would enable researchers to distinguish between initial implementation challenges and sustained intervention outcomes. Consequently, one of the strengths of this study is its demonstration that time should be considered an active component of leadership intervention effectiveness rather than merely a methodological consideration.

Several limitations should be considered when interpreting these findings. The study was conducted across three residential aged care facilities within a single organisation, which may limit transferability to other providers and care contexts. Participant numbers were relatively small and varied across data collection points. Mid‐trial observations were also conducted at only one of the three sites. Consequently, the observed difference in leadership time use should be interpreted descriptively rather than as within‐person change. The absence of a control group limits causal attribution of observed changes to the dyad house leadership model. Organisational indicators were based on a small number of paired observations, resulting in limited statistical power and requiring cautious interpretation of the inferential findings. In addition, residents and families were not involved in the later interpretive reflective process, which may have limited opportunities to further interrogate these perspectives. Longer‐term evaluation across multiple organisations and settings is required to establish the sustainability and transferability of the findings.

Notwithstanding these limitations, this study contributes novel evidence that dyad house leadership models can be successfully adapted to residential aged care and that its benefits extend beyond those previously reported in hospital‐based research. In particular, the study identifies role clarity, workforce pipeline perceptions and household‐level collaboration as important mechanisms influencing implementation and outcomes. These findings provide important new knowledge regarding how dyad leadership structures can support workforce sustainability, resident‐centred care and organisational transformation within contemporary aged care settings.

## 5. Implication for Nursing Management

The successful implementation of the dyad house leadership model highlights the value of distributing leadership responsibilities across both clinical and nonclinical roles. Organisations could consider leadership structures that enable responsibilities to be shared according to expertise, allowing clinical leaders to focus on care quality, professional practice and workforce development while nonclinical leaders address operational, environmental and administrative demands. This is particularly salient in environments such as residential aged care, which has experienced increasing clinical oversight and bureaucracy and a high clinical turnover environment, as such a model allows clinicians to focus on their strengths whilst releasing them from roles that take away time from clinical oversight. These arrangements may enhance leadership visibility, responsiveness and decision‐making capacity while reducing duplication of effort and leadership burden.

Furthermore, the findings of this study suggest that clearly defined responsibilities, decision‐making authority and reporting relationships are fundamental to the success of dyad leadership models. Nursing managers implementing similar approaches should invest in comprehensive role descriptions, communication strategies and ongoing role negotiation processes to minimise ambiguity and support collaboration between leadership partners. Moreover, investing in ongoing intra‐ and interprofessional team development is essential to ensure cohesive and harmonious teamwork between the dyads.

Finally, while the dyad house leadership model in this study generated positive outcomes, concerns relating to clinical authority, professional identity and accountability emerged during implementation. Nursing managers, therefore, need to establish clear governance and communication structures that articulate the boundaries of clinical and nonclinical decision‐making responsibilities while maintaining nursing accountability for resident care outcomes.

The findings also highlight the importance of considering succession planning and leadership pipelines when implementing dyad leadership models. Creating nonclinical leadership pathways may broaden the pool of employees able to move into management roles; however, organisations should ensure that this does not inadvertently narrow progression opportunities for nurses and other clinical staff. Nursing managers should, therefore, establish transparent clinical and nonclinical pathways, clarify how employees can progress into senior leadership roles and incorporate succession planning into the design of the dyad structure. This may help organisations diversify their future leadership pipeline while preserving meaningful advancement opportunities for clinicians.

## 6. Conclusion

In conclusion, this study provides the first empirical evidence that a dyad house leadership model can be successfully implemented within Australian residential aged care and can positively influence outcomes for staff, residents and families. By pairing clinical and nonclinical leaders within household units, the model improved role clarity, redistributed workloads, increased leadership visibility and enabled leaders to spend more time supporting staff and engaging with residents. These changes were associated with improvements in staff wellbeing, work‐life balance, job satisfaction and perceptions of care quality, while also enhancing residents’ and families’ experiences of communication, trust, continuity and overall satisfaction with care. From a workforce perspective, the model offers a promising strategy for addressing ongoing aged care workforce shortages through the creation of broader leadership pathways, improved role sustainability and enhanced staff retention. At the same time, the findings reinforce the importance of evaluating leadership interventions using outcomes that extend beyond workforce and organisational metrics to include the experiences of residents and families.

Future research should explore the long‐term sustainability and effectiveness of dyad house leadership models across diverse residential and community aged care settings. Longitudinal studies are needed to examine the extent to which improvements in workforce wellbeing, leadership effectiveness, resident outcomes and organisational performance are maintained over time. Further investigation of workforce pipeline outcomes, clinical leadership succession and household‐level collaboration would also strengthen the understanding of the mechanisms underpinning successful implementation. Finally, economic evaluations are warranted to determine the cost‐effectiveness and scalability of dyad leadership models within increasingly resource‐constrained and highly regulated aged care environments.

Overall, this study demonstrates that dyad house leadership represents a promising and innovative leadership approach for residential aged care. When supported by clear governance, role clarity, succession planning and adequate implementation timeframes, the model has the potential to strengthen workforce sustainability, improve care experiences and enhance quality of life for residents and their families.

## Funding

ARIIA, GA00198. Open access publishing was facilitated by Griffith University, as part of the Wiley–Griffith University agreement via the Council of Australasian University Librarians.

## Disclosure

The findings of this project have been previously presented at conferences and in industry publications. In addition, the dyad house leadership model has just won the 2026 You are ACE! Award in the Organisation‐Based‐Excellence category.

## Ethics Statement

Ethics was granted through Griffith University Human Research Ethics Committee. HREC/2023/335 & HREC/2023/336.

## Conflicts of Interest

The authors declare no conflicts of interest.

## Data Availability

The data that support the findings of this study are available on request from the corresponding author. The data are not publicly available due to privacy or ethical restrictions.
